# CCAAT/enhancer binding protein β is required for satellite cell self-renewal

**DOI:** 10.1186/s13395-016-0112-8

**Published:** 2016-12-07

**Authors:** Neena Lala-Tabbert, Hamood AlSudais, François Marchildon, Dechen Fu, Nadine Wiper-Bergeron

**Affiliations:** 1 Graduate Program in Cellular and Molecular Medicine, Faculty of Medicine, University of Ottawa, 451 Smyth Road, Ottawa, Ontario K1H 8M5 Canada; 2 Department of Cellular and Molecular Medicine, Faculty of Medicine, University of Ottawa, 451 Smyth Road, Ottawa, Ontario K1H 8M5 Canada

**Keywords:** C/EBPβ, Satellite cell, Self-renewal, Pax7, MyoD, Notch

## Abstract

**Background:**

Postnatal growth and repair of skeletal muscle relies upon a population of quiescent muscle precursor cells, called satellite cells that can be activated to proliferate and differentiate into new myofibers, as well as self-renew to replenish the satellite cell population. The balance between differentiation and self-renewal is critical to maintain muscle tissue homeostasis, and alterations in this equilibrium can lead to chronic muscle degeneration. The transcription factor CCAAT/enhancer binding protein beta (C/EBPβ) is expressed in Pax7^+^ satellite cells of healthy muscle and is downregulated during myoblast differentiation. Persistent expression of C/EBPβ upregulates Pax7, inhibits MyoD, and blocks myogenic differentiation.

**Methods:**

Using genetic tools to conditionally abrogate C/EBPβ expression in Pax7^+^ cells, we examined the role of C/EBPβ in self-renewal of satellite cells during muscle regeneration.

**Results:**

We find that loss of C/EBPβ leads to precocious differentiation at the expense of self-renewal in primary myoblast and myofiber cultures. After a single muscle injury, C/EBPβ-deficient satellite cells fail to self-renew resulting in a reduction of satellite cells available for future rounds of regeneration. After a second round of injury, muscle regeneration is impaired in C/EBPβ conditional knockout mice compared to wild-type control mice. We find that C/EBPβ can regulate Notch2 expression and that restoration of Notch activity in myoblasts lacking C/EBPβ prevents precocious differentiation.

**Conclusions:**

These findings demonstrate that C/EBPβ is a novel regulator of satellite cell self-renewal during muscle regeneration acting at least in part through Notch2.

## Background

Skeletal muscle has a remarkable capacity to regenerate after injury, which depends on the presence of muscle progenitor cells, called satellite cells (SC) [1, 2]. In adult muscle, satellite cells make up 2–7% of all myonuclei and express the paired-box transcription factor Pax7 [3–6]. After myofiber damage, SCs quickly upregulate the myogenic regulatory factor MyoD and re-enter the cell cycle to give rise to a population of transient-amplifying cells [7–9]. Subsequently, they downregulate Pax7, irreversibly withdraw from the cell cycle, upregulate myogenin expression, commit to terminal differentiation, and fuse to repair damaged fibers or to create new ones [8, 10–12]. While a large majority of myoblasts will differentiate into myocytes, a small percentage of myoblasts escape differentiation by maintaining Pax7 expression, downregulating MyoD expression, and returning to mitotic quiescence [1, 13]. As such, the expression of Pax7 and MyoD allows for classification of SCs as self-renewing (Pax7^+^/MyoD^−^), proliferating (Pax7^+^/MyoD^+^), or differentiating (Pax7^−^/MyoD^+^) [11, 13–15]. Self-renewal is important for maintaining the precursor pool and transplantation of SCs, or single muscle fibers can contribute to the repair of hundreds of new muscle fibers [1, 16] and replenishment of the stem cell niche [17].

CCAAT/enhancer binding proteins (C/EBPs) are a family of transcription factors that regulate cellular growth and differentiation, metabolism, and inflammation [18]. In skeletal muscle, C/EBPβ expression is localized to Pax7^+^ SCs of healthy muscle and is rapidly downregulated upon induction to differentiate [19, 20]. In vivo, loss of C/EBPβ expression in SCs resulted in larger muscle fiber cross-sectional area, fewer fibers, and improved repair after a single acute muscle injury [19, 20]. When overexpressed in the murine myoblast cell line C2C12 or in primary myoblasts, C/EBPβ reduced MyoD and other myogenic protein levels during differentiation, in addition to reducing myoblast fusion. C/EBPβ also increased Pax7 protein expression suggesting that C/EBPβ acts to maintain myogenic progenitors in an undifferentiated state [19, 21].

In this study, we examined the role of C/EBPβ in SC self-renewal using conditional knockout mice (*Cebpb* ^*fl/fl*^ *Pax7* ^*CreER/+*^) in which C/EBPβ is knocked down in Pax7^+^ cells following activation of the CreER recombinase with tamoxifen [19–23]. Loss of C/EBPβ in SCs results in precocious differentiation at the expense of self-renewal in both primary myoblasts and myofiber cultures. Further, C/EBPβ-deficient SCs are unable to self-renew after muscle injury leading to a reduction in the SC pool such that it cannot efficiently support muscle regeneration after a second round of injury. Mechanistic studies revealed that C/EBPβ can regulate Notch2 expression and self-renewal can be rescued in C/EBPβ-deficient myoblasts by overexpression of the Notch intracellular domain (NICD) indicating that C/EBPβ acts, at least in part, by regulating Notch signaling. Taken together, these results establish C/EBPβ as a novel regulator of SC homeostasis.

## Methods

### Mice and animal care

All animal work was performed in accordance with the guidelines set out by the Canadian Council on Animal Care and was approved by the University of Ottawa Animal Care Committee. A mouse bearing a C/EBPβ-floxed allele (C/EBPβ^fl/fl^) [22] was crossed with mice bearing the Pax7-CreER^tm^ allele [23] to generate C/EBPβ^fl/fl^Pax7^+/+^ (wild-type, WT) and conditional null C/EBPβ^−/−^Pax7^CreER−/+^ (C/EBPβ^−/−^) animals as previously described [19]. All animals were housed in a controlled facility (22 °C with 30% relative humidity on a 12-h light/dark cycle) and provided with food and water ad libitum. In vivo induction of CreER^tm^ activity, for myofiber and muscle injury experiments, was accomplished by performing daily intraperitoneal injections of 3 mg/40 g body weight of tamoxifen (dissolved in corn oil; Sigma-Aldrich) for 5 days.

For single BaCl_2_ injury, the mice were anesthetized with isoflurane and the hind limbs were shaved before the procedure. The C/EBPβ^fl/fl^ and C/EBPβ^−/−^Pax7^CreER−/+^ mice aged 8–10 weeks were injected with 50 μl of 1.2% BaCl_2_ in PBS into the left tibialis anterior (TA) muscle. The mice were sacrificed 4, 7, and 42 days post injury (dpi) and the TA was collected, embedded in Tissue-Tek OCT compound, flash frozen in isopentane cooled by liquid nitrogen, and sectioned (8-μm thick) for immunofluorescence. For double BaCl_2_ injury, the mice were allowed to recover from a BaCl_2_ injury for 21 days before a second injury to the same TA. The mice were sacrificed 7 and 21 days after the second injury.

### Primary and C2C12 myoblast culture

Primary myoblasts were isolated as described previously [24]. Briefly, the hind limb muscles of adult (6 to 8 weeks of age) mice were dissected and digested with collagenase/dispase (Roche). After digestion, the muscle slurry was filtered through a 70-μM cell strainer to remove undigested muscle. The cells were washed with serum-free media and then enriched for myoblasts by magnetic-activated cell sorting (MACS) [24]. The primary myoblasts were grown on matrigel-coated plates in growth media (GM: DMEM (Wisent) containing 20% FBS, 10% HS (Invitrogen) with penicillin and streptomycin (Wisent)) supplemented with 10 ng/ml basic fibroblast growth factor (bFGF) and 2 ng/ml human growth factor (HGF) (Peprotech). Differentiation was induced by changing the media of confluent cultures to differentiation media (DM: DMEM containing 2% FBS and 10% HS) for 48 h. To induce CreER^tm^ activity in culture, primary myoblasts from wild-type and conditional knockout animals were treated with 4-OH tamoxifen (2 μM dissolved in 100% ethanol; Sigma-Aldrich) for 48 h. To induce Notch activity, primary myoblasts were retrovirally transduced with empty vector (pLPCX) to express the Notch1 intracellular domain (pLPCX-NICD) and maintained in growth medium.

C2C12 murine myoblasts were retrovirally transduced with empty vector (pLXSN) ﻿or﻿ to express C/EBPβ (pLXSN-C/EBPβ), selected based on G418 resistance, and maintained in growth medium (DMEM with 10% FBS).

### Western analysis

Whole-cell extracts from primary myoblasts were resolved on a 12% SDS-PAGE gel, transferred to a PVDF membrane, and probed with specific antibodies: C/EBPβ (E299; Abcam), Notch intracellular domain 1 (NICD1; EMD Millipore), and cyclophilin B (Abcam). Chemiluminescence was detected with the ChemiDocTM MP System (Bio-Rad Laboratories).

### Limited trypsinization

Myotubes were separated from reserve cells in differentiated WT and C/EBPβ^−/−^ myoblast cultures as previously described [25]. Briefly, myoblasts were plated on 10-cm culture plates, cultured in GM until confluent, and then induced to differentiate in DM for 48 h. Following differentiation, myotubes were removed from reserve cells by limited trypsinization (0.15% trypsin for 5 min). Reserve cells, which remained attached to the culture plate, were removed with 0.25% trypsin. Reserve cells were counted and then re-plated to be induced for differentiation.

### Isolation and culture of single EDL myofibers

Myofibers were isolated from the extensor digitorum longus (EDL) muscle as described previously [26]. Briefly, the EDLs were removed from adult (6–8 weeks of age) mice and digested with collagenase type I (2 mg/ml in DMEM; Sigma-Aldrich). The muscles were transferred to horse serum-coated plates, and myofibers were separated by trituration using heat-polished glass Pasteur pipettes. Fibers were incubated for 72 h in DMEM supplemented with 15% FBS and 2% chick embryo extract at 37 °C, 5% CO_2_.

### Immunofluorescence

Myofibers were fixed in 4% paraformaldehyde (PFA) in PBS and 1% glycine and blocked in PBS containing 0.2% Triton X-100 (BioShop), 2% BSA, 5% goat serum (Cedarlane), and 1% azide. Myoblasts were fixed in 2% PFA in PBS and blocked in PBS containing 0.3% Triton X-100 and 10% goat serum. Cryosections were thawed at room temperature, fixed in 4% PFA, and processed for antigen retrieval in citrate buffer at 95 °C for 20 min. The sections were permeabilized with PBS containing 0.5% Triton X-100 and blocked in PBS containing 0.1% Triton X-100 and 5% donkey serum (Cedarlane) prior to incubation with primary antibody overnight at 4 °C. The cells were washed with PBS and incubated in biotin anti-mouse (when indicated) or secondary antibodies conjugated to a fluorescent dye (Cy3, Alexa 488, or Alexa Flour 647; all from Jackson ImmunoResearch). Nuclei were counterstained with DAPI (0.5 μg/ml). The primary antibodies used were as follows: Pax7-c (DSHB), MYH (H-300; Santa Cruz), MyoD (C-20; Santa Cruz), myogenin (M-225; Santa Cruz), C/EBPβ (E299; Abcam), and laminin (AL-4; Millipore).

### Image acquisition

Digital images of the stained myoblasts, myofibers, and muscle sections were acquired at room temperature using a microscope (Leica DM 3000B), Infinity-3 camera (Lumenera), and Infinity Capture imaging software (Lumenera). The images were composed and edited in paint.net.

### Chromatin immunoprecipitation

C2C12 myoblasts were crosslinked for 30 min at room temperature with 1% formaldehyde and sonicated with Diagenode bioruptor®. Chromatin immunoprecipitation (ChIP) analysis was performed as described [27] using antibodies against C/EBPβ (C-19; Santa Cruz) or rabbit IgG as negative control. Protein G conjugated Dynabeads (Invitrogen) were used to precipitate immunoconjugates, and DNA fragments were purified using the QIAquick PCR purification kit (Qiagen). A standard curve was generated using input DNA for each immunoprecipitate, and quantification was determined as the percentage of enrichment relative to 10% input for each condition. Primer sequences for qPCR amplification are as follows: *Notch2* R1 (–19 kb; chr3:97,797,949-97,798,354) F: TGAGGAAGTTGACAGGGAGC, R: GTGTCCAGGGCAACTTGGAA; *Notch2* R2 (+11 kb; chr3:97,828,475-97,828,573) F: GGAAGCGATCGGTGTTGTTG, R: AAAAGCAGTGGGGCGTCTTA; *Notch2* R3 (+14 kb; chr3:97,831,399-97,831,590) F: TAGGAAGGATGTGGGGAGGG, R: ATCTGACACAGCAGCTTCCC; and *Notch2* R4 (+43 kb; chr3:97,860,912-97,861,087) F: CTTCGTCCCTCAACCTCCTG, R: AATAGGGCCGTGGCAGAAAA.

### Statistical analysis

Statistical analysis was performed using GraphPad Prism software (GraphPad Software, La Jolla, CA, USA). A student’s *t* test was used when comparing a single experimental condition to the control condition. A one-way ANOVA was performed when comparing three or more experimental conditions. A Tukey’s post hoc test was used when the ANOVA was significant. The cutoff for significance was *p* < 0.05. All experiments are representative of a minimum of three biological replicates, and data is presented as mean ± standard error mean (SEM).

## Results

### C/EBPβ-deficient satellite cells display increased differentiation

**Fig. 1:**
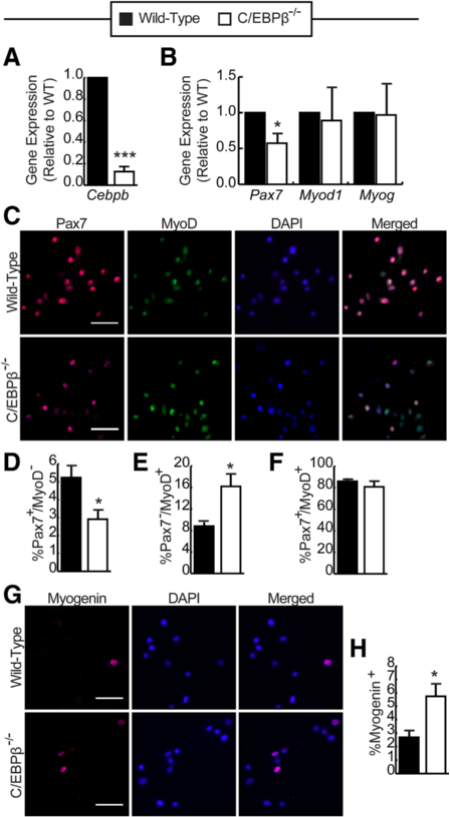
Loss of C/EBPβ reduces the proportion of Pax7^+^ cells in myogenic cultures*.* **a** RT-qPCR analysis of *Cebpb* expression in primary myoblasts isolated from C/EBPβ^fl/fl^Pax7^+/+^ (wild-type) and conditional null C/EBPβ^−/−^Pax7^CreER−/+^ (C/EBPβ^−/−^) subjected to in vitro 4′OH-TAM treatment and cultured in growth medium (GM) (*n* = 5). **b** RT-qPCR analysis of *Pax7*, *Myod1*, and *Myog* expression in primary myoblasts isolated and cultured as in **a** (*n* = 5). **c** Representative pictures of myoblasts cultured under growth conditions for 24 h stained for Pax7 (*red*) and MyoD (*green*). Nuclei were counterstained with DAPI (*blue*). *Scale bar*, 50 μm. Percentage of **d** self-renewing (Pax7^+^/MyoD^−^), **e** differentiating (Pax7^−^/MyoD^+^), and **f** proliferating (Pax7^+^/MyoD^+^) WT (*black bars*) and C/EBPβ^−/−^ (*white bars*) myoblasts cultured as in **a** as determined by immunocytochemistry (*n* = 4). **g** Representative pictures of cells cultured as in **a** and immunostained for myogenin (*red*). The nuclei were counterstained with DAPI (*blue*). *Scale bar*, 50 um. **h** Percentage of myogenin^+^ cells relative to total nuclei (*n* = 4). For all panels, data is represented as mean ± SEM, *p < 0.05, ***p < 0.001

To investigate the functional consequences of disrupting C/EBPβ expression in SCs, we isolated SCs from conditional knockout mice (*Cebpb* ^*fl/fl*^ *Pax7* ^*CreER/+*^) in which C/EBPβ expression is abrogated in Pax7^+^ cells following activation of the CreER recombinase with tamoxifen. SC-derived myoblasts (*Cebpb* ^*fl/fl*^ *Pax7* ^*+/+*^ (WT) and C/EBPβ^−/−^) were cultured in high serum (GM) for 24 h, and excision was confirmed by RT-qPCR, with *Cebpb* expression reduced to ~10% of controls (Fig. 1a). In C/EBPβ^−/−^ cells, *Pax7* expression was significantly reduced by approximately 50% as compared to the WT controls, while *Myod1* and *Myog* expression were unaffected (Fig. 1b). As C/EBPβ is a known regulator of Pax7 and MyoD protein expression, the proportion of self-renewing (Pax7^+^/MyoD^−^), proliferating (Pax7^+^/MyoD^+^), and differentiating (Pax7^−^/MyoD^+^) cells was assessed in WT and C/EBPβ^−/−^ myoblasts in growth medium by immunostaining (Fig. 1c) [19]. The percentage of Pax7^+^/MyoD^−^ (self-renewing) cells was decreased in C/EBPβ^−/−^ myoblasts compared to WT myoblasts (2.9 vs. 5.2%) with a concomitant increase in Pax7^−^/MyoD^+^ differentiating cells (16.2 vs. 8.8%) (Fig. 1d, e). There was no significant change in the population of Pax7^+^/MyoD^+^ proliferating cells between C/EBPβ^−/−^ myoblasts and WT myoblasts in GM (Fig. 1f).

Given the larger population of Pax7^−^/MyoD^+^ cells in C/EBPβ^−/−^ cultures, we assessed myogenin expression by immunostaining. While no significant differences were observed in mRNA expression over the population (Fig. 1b), there was a significant ~2-fold increase in the percentage of myogenin-positive cells in C/EBPβ^−/−^ myoblast cultures as compared to WT (Fig. 1g, h) suggesting that C/EBPβ-deficient cells are more predisposed to precociously differentiate under growth conditions, consistent with our previous findings [19].

**Fig. 2:**
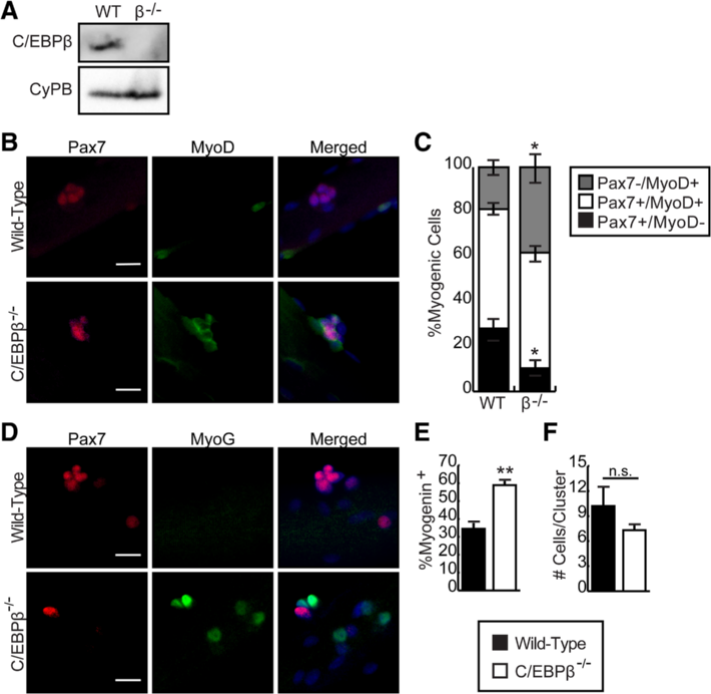
Loss of C/EBPβ inhibits satellite cell self-renewal and promotes differentiation. C/EBPβ^fl/fl^Pax7^+/+^ (wild-type) and conditional null C/EBPβ^−/−^Pax7^CreER−/+^ (C/EBPβ^−/−^) mice were injected daily with tamoxifen for 5 days. One week after the last injection, myofibers were isolated from the EDL of each mouse and cultured for 3 days in suspension before immunostaining. **a** Western analysis of C/EBPβ expression in SCs isolated from the hind limb of WT and C/EBPβ^−/−^ mice injected with tamoxifen for 5 days. **b** Representative pictures of myofibers stained for Pax7 (*red*) and MyoD (*green*). Nuclei were counterstained with DAPI (blue). *Scale bar*, 20 μm. **c** Quantification of self-renewing (Pax7^+^/MyoD^−^), proliferating (Pax7^+^/MyoD^+^), and differentiating (Pax7^−^/MyoD^+^) myoblasts on single myofibers (*n* = 4 mouse pairs). **d** Representative pictures of myofibers stained for Pax7 (*red*) and myogenin (*green*). Nuclei were counterstained with DAPI (*blue*). *Scale bar*, 20 μm. **e** Quantification of myogenin^+^ cells as a percentage of total nuclei (*n* = 3 mouse pairs). **f** Total number of cells per cluster (*n* = 3 mouse pairs). For all panels, data is represented as mean ± SEM, *p < 0.05, **p < 0.01, *n.s.* not significant

To further investigate C/EBPβ-dependent alteration in cell fate choices, we used single myofiber preparations isolated from the EDL muscles of wild-type (WT) and conditional null (C/EBPβ^−/−^) animals 1 week after daily tamoxifen (3 mg/40 g body weight for 5 days) injections to induce excision. C/EBPβ excision was confirmed by western blot of cell lysates from satellite cells isolated from the hind limb muscles (except EDL) of the tamoxifen-treated animals (Fig. 2a). Myofibers from the WT and C/EBPβ^−/−^ mice were immunostained for Pax7 and MyoD and counterstained with DAPI to determine the number of self-renewing (Pax7^+^/MyoD^−^), activated (Pax7^+^/MyoD^+^), and differentiated (Pax7^−^/MyoD^+^) SCs 72 h after isolation. The myofibers from the C/EBPβ^−/−^ animals had significantly fewer Pax7^+^/MyoD^−^ SCs (10.4%) and more Pax7^−^/MyoD^+^ SCs (38.3%) compared to WT EDL myofibers (28.1 and 18.9%, respectively) without affecting the proliferating Pax7^+^/MyoD^+^ population (Fig. 2b, c). Furthermore, when immunolabeled for Pax7 and myogenin, the myofibers from the C/EBPβ^−/−^ animals had significantly more myogenin^+^ cells (59%) compared to the control EDL myofibers (34%) (Fig. 2d, e). There were no changes in the number of satellite cells per cluster on WT and C/EBPβ^−/−^ myofibers, confirming that loss of C/EBPβ does not inhibit SC activation (Fig. 2f). These results further demonstrate that C/EBPβ promotes SC self-renewal and that loss of C/EBPβ in SCs leads to increased differentiation.

### C/EBPβ is required for the generation of reserve cells in vitro

**Fig. 3:**
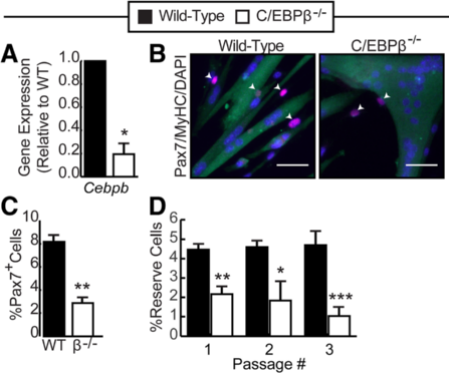
Loss of C/EBPβ leads to a reduction in reserve cells in vitro. **a** RT-qPCR analysis of *Cebpb* expression in primary myoblasts isolated from C/EBPβ conditional knockout mice (C/EBPβ^−/−^) or wild-type non-Cre-expressing littermates subjected to in vitro 4’OH-TAM treatment, cultured in GM, and plated and transferred to differentiation media for 48 h (*n* = 3). **b** Representative pictures of cells stained for myosin heavy chain (MyHC; *green*) and Pax7 (*red*). Nuclei were counterstained with DAPI (*blue*). *Scale bar*, 50 μm. **c** Percentage of Pax7^+^ cells relative to total nuclei (*n* = 3). **d** Reserve cells were collected and counted following limited trypsinization after 48 h in differentiation media. After counting, the reserve cells were expanded, re-plated at equal densities, and induced to differentiate in low serum for another 48 h. The number of reserve cells was counted after each round of differentiation and represented as a percentage of total nuclei (*n* = 3). Data is represented as mean ± SEM, *p < 0.05, **p < 0.01, ***p < 0.001

To examine the role of C/EBPβ in SC self-renewal during myogenic differentiation, SC-derived myoblasts (wild-type and C/EBPβ^−/−^) were cultured in high serum (GM) for 24 h and then switched to low serum (DM) for 48 h to induce differentiation. RT-qPCR analysis confirmed excision of *Cebpb* in the myoblasts isolated from the C/EBPβ^−/−^ animals (Fig. 3a). Terminally differentiated cultures were fixed and stained for myosin heavy chain (MyHC) and Pax7 expression to quantify myoblast fusion and reserve cell formation, respectively (Fig. 3b) [25, 28]. The differentiated C/EBPβ-null cultures had fewer Pax7^+^ reserve cells (2.8%) compared to the control cultures (8.2%) (Fig. 3b, c) consistent with the reduction of Pax7^+^/MyoD^−^ cells observed in GM (Fig. 1d). Pax7^+^ reserve cells can be separated from myotubes by limited trypsinization and differentiated again to study self-renewal dynamics in a cell population [25, 28]. To determine whether C/EBPβ^−/−^ reserve cells function normally, we collected reserve cells by limited trypsinization and, after expansion and equal plating, induced them to differentiate. After each round of differentiation, we counted the total number of reserve cells. After each passage, despite equal plating, there were fewer C/EBPβ^−/−^ reserve cells compared to wild-type cells (Fig. 3d). Further, after each round of differentiation, the number of reserve cells trended towards a decrease in the C/EBPβ^−/−^ cultures but did not achieve statistical significance (Fig. 3d). These data suggests that C/EBPβ is required for maintenance of reserve cells in vitro.

### C/EBPβ is required to restore the muscle satellite cell pool during regeneration

**Fig. 4:**
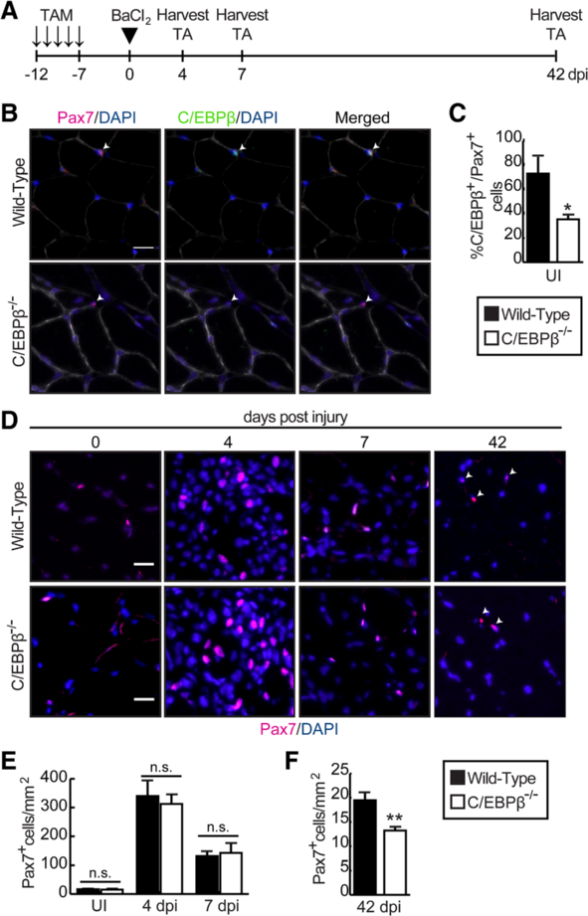
C/EBPβ is required for restoring the muscle stem cell pool after acute injury. **a** Experimental design. C/EBPβ^fl/fl^ Pax7^+/+^ (wild-type) and conditional null C/EBPβ^−/−^Pax7^CreER−/+^ (C/EBPβ^−/−^) mice were injected daily with tamoxifen for 5 days. One week after the last injection, the left tibialis anterior (TA) muscle was injured by intramuscular injection of 50 μl of 1.2% BaCl_2_. The muscle was analyzed at 0, 4, 7, and 42 days post injury (dpi). **b** Cross-sections of uninjured TA were immunostained for Pax7 (*red*), C/EBPβ (*green*), and laminin (*white*). Nuclei were counterstained with DAPI (*blue*). Representative pictures are shown. *Scale bar*, 20 μm. **c** Excision efficiency in Cre^+^ satellite cells represented by the percentage of C/EBPβ^+^ nuclei among total Pax7^+^ cells in uninjured muscle (*n* = 3). **d** Cross-sections of injured TA 0, 4, 7, and 42 dpi were immunostained for Pax7 (*red*) and counterstained with DAPI (*blue*). Representative pictures are shown. *Scale bar*, 20 μm. **e** Pax7^+^ cells per area (mm^2^) at 0, 4, and 7 dpi (*n* ≥ 3 mouse pairs). **f** Pax7^+^ cells per area (mm^2^) at 42 dpi (*n* = 4 mouse pairs). For all panels, data is represented as mean ± SEM, *p <0.05,﻿﻿ **p < 0.01, *n.s.* not significant

To confirm the reduced self-renewal potential of C/EBPβ^−/−^ satellite cells in vivo, an acute injury was induced by BaCl_2_ injection into the TA muscle of the C/EBPβ^fl/fl^Pax7^+/+^ (wild-type) and conditional null C/EBPβ^−/−^Pax7^CreER−/+^ (C/EBPβ^−/−^) animals 1 week after tamoxifen administration (3 mg/40 g body weight daily for 5 days). The TA muscles were collected 0, 4, 7, and 42 dpi (Fig. 4a). Excision was confirmed by dual immunofluorescence staining of TA muscle sections for Pax7 and C/EBPβ (Fig. 4b, c). Immunostaining of injured TA muscle at 0, 4, and 7 dpi for Pax7 revealed that there were no changes in the number of Pax7^+^ cells in the C/EBPβ-deficient muscle compared to the wild-type muscle (Fig. 4d, e), suggesting that C/EBPβ-deficient myoblasts expand efficiently after muscle injury.

During postnatal regeneration, the size of the Pax7^+^ satellite cell pool reaches homeostatic levels observed in the uninjured muscle 40 days after injury [29]. In the wild-type muscle, Pax7^+^ cell numbers returned to non-injured levels; however, there were significantly fewer Pax7^+^ cells in C/EBPβ-null muscle (Fig. 4d, f). These findings suggest that C/EBPβ is essential for directing cells towards self-renewal after injury.

### Loss of C/EBPβ expression cripples the long-term regenerative response

**Fig. 5:**
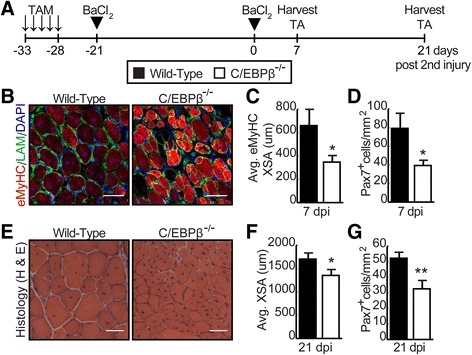
Loss of C/EBPβ expression in satellite cells decreases regenerative capacity following serial injury. **a** Experimental design. C/EBPβ^fl/fl^ Pax7^+/+^ (wild-type) and conditional null C/EBPβ^−/−^Pax7^CreER−/+^ (C/EBP β^−/−^) mice were injected daily with tamoxifen for 5 days. One week after the last injection, the left tibialis anterior (TA) muscle was injured using 50 μl of 1.2% BaCl_2_ (1st injury). Twenty-one days after the initial injury, the same TA muscle was injured again using 50 μl of 1.2% BaCl_2_ (2nd injury). The muscle was analyzed at 7 and 21 days after the 2nd injury (dpi). **b** Cross-sections of injured TA 7 dpi were stained with embryonic myosin heavy chain (eMyHC; *red*) and laminin (LAM; *green*). Nuclei were counterstained with DAPI (*blue*). Representative pictures are shown. *Scale bar*: 50 μm. **c** Average cross-sectional area of eMyHC^+^ fibers from TA sections stained as in **b** (*n* = 5 mouse pairs). **d** Pax7^+^ nuclei were counted in 7 dpi muscle and represented as total Pax7^+^ cells per area (mm^2^) (*n* = 4 mouse pairs). **e** Cross-sections of injured TA 21 dpi were stained for hematoxylin (nuclei; *purple*) and eosin (*pink*) (H&E). Representative pictures are shown. *Scale bar*, 50 μm. **f** Average cross-sectional area of myofibers from TA sections stained as in **e** (*n* = 6 mouse pairs). **g** Pax7^+^ nuclei were counted in 21 dpi muscle and represented as total Pax7^+^ cells per area (mm^2^) (*n* = 3 mouse pairs). Data is represented as mean ± SEM, *p < 0.05, **p < 0.01

As SC self-renewal is essential for efficient repair of tissue after injury, we examined muscle regeneration in wild-type and conditional null (C/EBPβ^−/−^) animals after two serial BaCl_2_ injuries separated by 21 days. TA muscles were collected 7 and 21 days after the second injury for analysis (Fig. 5a). One week following double injury (7 dpi), the TA muscle sections were stained for embryonic myosin heavy chain (eMyHC) to measure the size of newly regenerated fibers. Contrary to what was observed after a single injury [19], the average cross-sectional area of eMyHC^+^ fibers was significantly smaller in the C/EBPβ^−/−^ muscle (348 μm) compared to the wild-type muscle (661 μm) (Fig. 5b, c). There were also fewer Pax7^+^ cells in the C/EBPβ-deficient muscle compared to the wild-type muscle (Fig. 5d). To confirm that the smaller fiber size in C/EBPβ-null animals was not due to a delay in regeneration, we assessed the average cross-sectional area of fibers 21 days after secondary injury (21 dpi). Consistent with our 7 dpi findings, muscle fibers from the C/EBPβ^−/−^ animals were smaller (1353 um) compared to the wild-type fibers (1703 μm) (Fig. 5e, f) and the percentage of Pax7^+^ SCs remained lower in C/EBPβ-deficient muscle (Fig. 5g). Taken together, these results strongly suggest that C/EBPβ is essential for SC self-renewal that is necessary for continual muscle regeneration.

### Activation of Notch signaling rescues self-renewal in C/EBPβ-deficient myoblasts

**Fig. 6:**
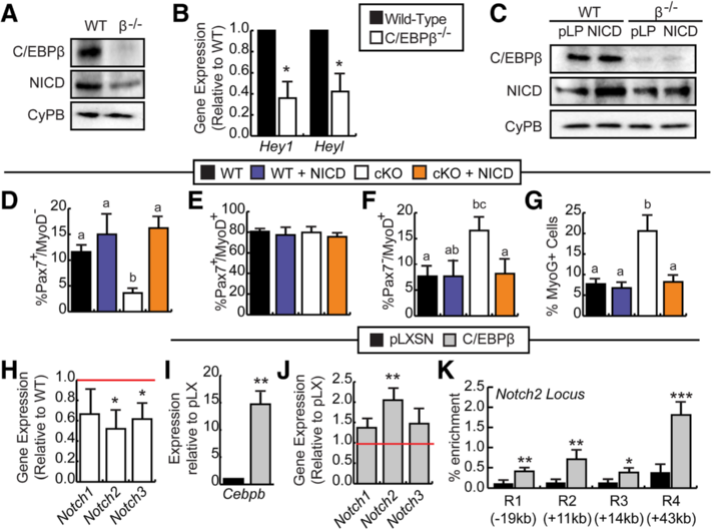
Activation of Notch signaling rescues self-renewal in C/EBPβ-deficient myoblasts. **a** Western analysis of C/EBPβ and NICD expression in primary myoblasts isolated from C/EBPβ conditional knockout mice (β^−/−^) or wild-type non-Cre-expressing littermates (WT) subjected to in vitro 4′OH-TAM treatment and cultured in growth medium (GM). Cyclophilin B (CyPB) is a loading control. **b** RT-qPCR analysis of *Hey1* and *Heyl* expression in primary myoblasts isolated and cultured as in **a** (*n* = 5). **c** C/EBPβ and Notch1 intracellular domain (NICD) protein expression in wild-type (WT) and C/EBPβ^−/−^ (cKO) primary myoblasts retrovirally transduced to express NICD or with empty virus (pLPCX; pLP) cultured in GM for 48 h (*n* = 3). Percentage of **d** self-renewing (Pax7^+^/MyoD^−^), **e** proliferating (Pax7^+^/MyoD^+^), and **f** differentiating (Pax7^−^/MyoD^+^) WT (*black bars*), WT + NICD (*blue bars*), C/EBPβ^−/−^ (*white bars*), and C/EBPβ^−/−^ + NICD (*orange bars*) myoblasts cultured as in **c** as determined by immunocytochemistry (*n* = 3). **g** Percentage of myogenin^+^ cells relative to total nuclei (*n* = 3). **h** RT-qPCR analysis of *Notch1*, *Notch2*, and *Notch3* expression in primary cells transduced to express C/EBPβ and cultured as in **a**, shown relative to WT controls indicated by the *red line* (*n* = 5). **i** *Cebpb* and **j** *Notch1*, *Notch2* and *Notch3* expression in C2C12s retrovirally transduced to express C/EBPβ relative to empty vector controls (pLXSN, *red line*) cultured in growth medium for 24 h (*n* = 4). **k** ChIP analysis of C/EBPβ occupancy at four regions (R1–R4) of the *Notch2* locus in C2C12s retrovirally transduced to express C/EBPβ or empty vector (pLXSN) in GM. RT-qPCR data is shown as the percent enrichment relative to 10% input used for immunoprecipitation for each condition (*n* = 4). Approximate region locations are relative to the transcriptional start site. Data is represented as mean ± SEM, *p < 0.05, **p < 0.01, ***p < 0.001. Means with *different letters* are significantly different

The Notch pathway is an established regulator of SC self-renewal and homeostasis [30–33]; thus, we examined whether the expression of Notch1 intracellular domain (NICD) and Notch target genes (*Hey1*, *Heyl*) were disrupted in C/EBPβ^−/−^ myoblasts grown in GM. Western analysis revealed that the amount of the activated Notch (NICD) was decreased in the C/EBPβ^−/−^ myoblasts as compared to the control myoblasts (Fig. 6a). Consistent with the reduced NICD, RT-qPCR revealed that the expression of the Notch target genes *Hey1* and *Heyl* was significantly downregulated in myoblasts deficient in C/EBPβ compared to wild-type myoblasts (Fig. 6b).

Since Notch signaling is reduced in C/EBPβ^−/−^ myoblasts and Notch signaling is known to inhibit myogenic differentiation, we examined whether precocious differentiation in these cells could be rescued by NICD overexpression. Western analysis confirmed C/EBPβ excision and NICD overexpression in WT and C/EBPβ^−/−^ myoblasts retrovirally transduced to express the NICD or with empty vector (pLP) (Fig. 6c). While the population of Pax7^+/^MyoD^−^ cells was significantly reduced with the loss of C/EBPβ expression, this population was restored to control levels with expression of the NICD (Fig. 6d). There were no significant changes in the Pax7^+^/MyoD^+^ population, but NICD overexpression prevented the precocious differentiation (Pax7^−^/MyoD^+^) observed in C/EBPβ^−/−^ myoblasts (Fig. 6e, f). Myogenin staining confirmed this finding (Fig. 6g), suggesting that the loss of self-renewal in C/EBPβ^−/−^ myoblasts is due to decreased Notch signaling.

To determine how C/EBPβ regulates Notch signaling, we assessed Notch receptor expression (*Notch1*, *Notch2*, and *Notch3*) in wild-type and C/EBPβ^−/−^ myoblasts, as well as, C2C12s retrovirally transduced to overexpress C/EBPβ or with empty vector (pLXSN). RT-qPCR analysis revealed that both *Notch2* and *Notch3* were significantly downregulated in C/EBPβ^−/−^ myoblasts in the growth medium, while *Notch1* expression was reduced but failed to reach statistical significance (Fig. 6h). In proliferating C2C12s overexpressing *Cebpb*, *Notch2* was the only Notch receptor significantly upregulated as compared to empty virus controls (Fig. 6i, j), suggesting that *Notch2* is regulated by C/EBPβ in myoblasts. We next examined whether C/EBPβ could occupy DNA regulatory elements controlling *Notch2* expression. Using available ChIP-seq data (GSE36024), we identified four peaks of C/EBPβ binding (E1–E4) of which E3 is a known *Notch2* enhancer [34] and assessed C/EBPβ occupancy of these regions by chromatin immunoprecipitation (Fig. 6k). Under growth conditions, we found that C/EBPβ could occupy all of the regions identified by ChIP-seq, suggesting that CEBPβ can control entry into differentiation and self-renewal, at least in part, through regulation of *Notch2* expression and Notch activity.

## Discussion

While most activated satellite cells will progress through myogenic differentiation, a small fraction escape, return to quiescence, and self-renew to maintain the muscle stem cell population, marked by loss of MyoD expression. In addition to downregulation of MyoD expression, it is known that the SC population itself is heterogeneous and can undergo both asymmetrical and symmetrical divisions in the context of the niche, with asymmetric distribution of cellular proteins and chromatin templates which regulate the balance between SC maintenance and regenerative potential. Analysis of myogenic regulatory factor (Myf5 and MyoD) expression in proliferating SCs revealed asymmetric expression of these factors in newly divided daughter cells [35–37]. These multiple mechanisms implicated in the maintenance of the skeletal muscle stem cell pool underlie the importance of these cells for muscle homeostasis. Too many cells destined towards repair results in rapid depletion of the SC compartment as observed with the loss of C/EBPβ, while enhanced self-renewal restrains regeneration, with both disequilibria leading to muscle atrophy. Recently, loss of dystrophin expression in SCs in Duchenne muscular dystrophy was shown to reduce the available pool of SCs for repair, contributing to the pathogenesis of this disease [38].

While self-renewal is important for maintaining the precursor pool, the mechanisms by which some SCs evade differentiation remain poorly understood. In this study, we identify C/EBPβ as a novel regulator of SC self-renewal, demonstrating that in its absence, Pax7^+^/MyoD^−^ cells are generated in lesser number, resulting in a reduction of the satellite cell pool. While muscle in the conditional nulls repairs with greater efficiency after a single injury due to an increased propensity to differentiate [19], this repair results in a significant decrease in the SC population that cannot efficiently support repair after a second injury. However, using our conditional model, we observed a reduction in the satellite cell pool, rather than depletion, attributable to the incomplete excision of *Cebpb* and therefore the contribution of recombination escapers.

C/EBPβ is normally downregulated in early differentiation, a step that is required for full expression of MyoD and differentiation to occur [19]. Indeed, loss of C/EBPβ expression does not affect the progression towards the Pax7^+^/MyoD^+^ state. It remains unclear whether C/EBPβ is asymmetrically distributed in proliferating myoblasts or whether its expression is re-initialized in a small population of Pax7^+^ cells destined to self-renew. Indeed, while C/EBPβ expression does not appear to be required for Pax7 expression [19], it can force Pax7 to be expressed under differentiation conditions, and this mechanism could prevent myoblasts from completing myogenic differentiation. Many studies have demonstrated that adult skeletal muscles lacking Pax7-expressing satellite cells cannot regenerate [2, 39, 40].

A number of signaling pathways have been implicated in SC self-renewal. Recent literature suggests that Notch signaling is crucial for SCs to self-renew and return to quiescence [30–33]. In adult muscle, the absence of Notch signaling induced spontaneous activation and differentiation of SCs which lead to a reduction in self-renewal and consequently impaired muscle regeneration [31, 32]. Furthermore, overexpression of Notch intracellular domain (NICD^OE^) upregulates Pax7, downregulates MyoD, and inhibits S-phase entry of primary myoblasts [33]. SC specific NICD^OE^ also impaired regeneration of skeletal muscle [33] and loss of downstream Notch target genes Hesr1 (Hey1) and Hesr3 (HeyL) led to precocious differentiation, reduced SC self-renewal, and a reduced SC pool [30]. Interestingly, we show that the loss of C/EBPβ in SC-derived myoblasts results in a significant downregulation in genes involved in the Notch signaling pathway (Fig. 6) suggesting one possible mechanism by which C/EBPβ regulates SC self-renewal is through regulation of Notch. Indeed, changes in C/EBPβ expression induced changes in both Notch receptor expression and Notch activity as measured by *Hey1* and *Heyl* expression. While we identify *Notch2* as a target of C/EBPβ activity, we demonstrate that the Notch1 intracellular domain is also reduced, suggesting that C/EBPβ may regulate the Notch pathway at multiple levels. Recently, over-activation of Notch signaling was shown in progenitor cells in skeletal muscle of dystrophic mice [41]. We have shown that C/EBPβ expression persists in SCs of cachectic muscles and that these cells do not differentiate [42, 43]; thus, it is tempting to hypothesize that C/EBPβ may persist in SCs of dystrophic muscle, upregulating Notch expression and contributing to the dystrophic defect. Our findings provide a mechanism for C/EBPβ-induced cell fate choices in myogenesis and identify C/EBPβ as a novel regulator of Notch signaling.

## Conclusions

C/EBPβ-deficient satellite cells are unable to efficiently self-renew after muscle injury, leading to a reduction in the SC pool and impaired regenerative capacity after serial injury. Taken together, our results establish C/EBPβ as a novel regulator of SC homeostasis that promotes differentiation at the expense of self-renewal. For example, while increased expression of C/EBPβ in cachectic muscle inhibits repair of wasting fibers, C/EBPβ protects the stem cells from apoptosis. The characterization of transcription factors controlling SC homeostasis provides important insights into the molecular mechanisms regulating skeletal muscle regeneration and further insight into the regulation of C/EBPβ function, and its biological role in the context of healthy and diseased muscle can fuel the development of novel therapeutic approaches for the treatment of muscle atrophies.

## Abbreviations

C/EBPβ: CCAAT/enhancer binding protein beta
ChIP: Chromatin immunoprecipitation
DM: Differentiation media
dpi: Days post injury
EDL: Extensor digitorum longus
eMyHC: Embryonic myosin heavy chain
GM: Growth media
IHC: Immunohistochemistry
mRNA: Messenger ribonucleic acid
MyHC: Myosin heavy chain
MyoD: Myogenic differentiation factor 1
MyoG: Myogenin
NICD: Notch intracellular domain
Pax7: Paired box protein 7
PBS: Phosphate-buffered saline
PFA: Paraformaldehyde
RT-qPCR: Real-time quantitative polymerase chain reaction
SC: Satellite cell
TA: Tibialis anterior
WT: Wild-type
